# Sono-chemical mechanism of simultaneous Green Malachite and Rhodamine B removal using blue crab shell biochar

**DOI:** 10.1016/j.ultsonch.2026.108054

**Published:** 2026-09-10

**Authors:** Najia Hamrouni, Najla Ben Ameur, Noureddine Allouche, Jean-Yves Hihn, Wafa Sassi

**Affiliations:** a Unit Modeling, Analysis and Control of Systems (MACS), National Engineering School of Gabes, Gabes, Tunisia; b Laboratory of Wastewaters and Environment, Center of Water Research and Technologies (CERTE) Technopark of Borj Cedria PB 273, Soliman 8020, Tunisia; c Laboratory of Organic Chemistry LR17-ES08 (Natural Substances Team), Faculty of Sciences of Sfax, University of Sfax, Sfax, Tunisia; d Université Marie et Louis Pasteur, CNRS Institut UTINAM UMR, 6213 Besançon, France

**Keywords:** CCD model, Crustacean waste, Batch adsorption, Ultrasonics

## Abstract

This study investigates the simultaneous removal of Malachite Green (GM) and Rhodamine B (RB) using blue crab shell biomass and its derived biochar. Thermal and textural characterizations revealed the superior stability and hierarchical microporous structure of biochar compared with biomass. CCD optimization identified a contact time of 79 min, an initial dye concentration of 95 mg L^−1^, and a temperature of 310 K as the optimal adsorption conditions, yielding removal efficiencies of 97.24 % and 94.31 % for GM and RB, respectively, using biochar, compared with 81.24 % and 76.31 % using biomass. Ultrasound-assisted adsorption was subsequently performed using a 40 kHz ultrasonic system at powers ranging from 50 to 300 W. Maximum cavitation activity was observed at 200 W, at which GM and RB removal reached 94.6 % and 92.3 % for biomass and 99.6 % and 99.0 % for biochar, respectively. Sono-chemiluminescence analysis confirmed enhanced acoustic cavitation at the optimum power. SEM observations further indicated improved mass transfer and more homogeneous dye distribution after ultrasonic treatment. A cavitation-based sonochemical mechanism involving microjets, shock waves, and microstreaming was proposed to explain the enhanced adsorption. These findings highlight the synergistic effect of acoustic cavitation and biochar for the efficient simultaneous removal of GM and RB from aqueous media.

## Graphical abstract

**Figure:**
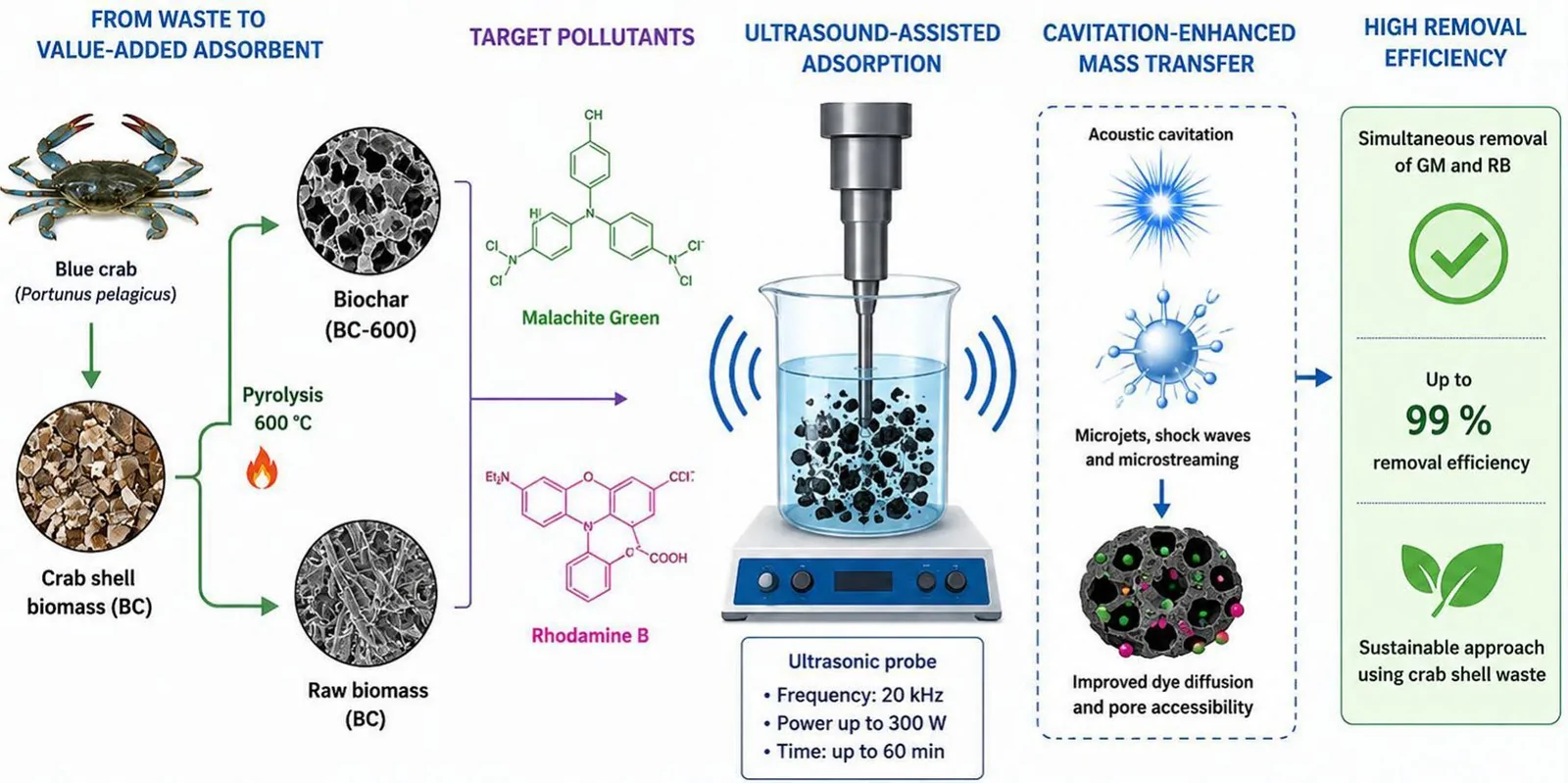

## 1 Introduction

Removal of organic dyes from wastewater is a major environmental challenge due to their persistence in aquatic environments and potential toxicity to ecosystems and human health [1]. Among the many dyes used in the textile and chemical industries, green malachite (GM) and rhodamine B (RB) are two of the most common, and their presence in wastewater requires effective solutions to prevent pollution and protect water resources [1], [2], [3]. Conventional water treatment methods, such as adsorption onto activated carbon, coagulation or filtration, although effective, present disadvantages such as high costs and limited regeneration capacity [1], [2], [3], [4]. These limitations have led to the search for alternative and sustainable materials for the adsorption of organic pollutants, and biochars derived from local biomasses, such as crustacean waste, have emerged as an innovative and cost-effective solution [5], [6], [7].

Biochars, produced by pyrolyzing organic materials, possess unique characteristics such as a large surface area, porous structure, and active sites that promote the adsorption of various organic pollutants, including dyes [8], [9]. In particular, crustacean waste, rich in chitosan and other organic compounds, has attracted increasing interest as a feedstock for biochar production due to its availability, low cost, and potential for treating contaminants in wastewater [9], [10], [11], [12]. Chitosan, a natural polymer found in crustacean shells, is known for its chelation properties and interaction with organic substances, making it a promising adsorbent for dyes [13], [14], [15].

However, despite the appeal of these bio-based materials, there is still a need to deepen the understanding of the specific adsorption mechanisms, optimal pyrolysis conditions, and biochar durability in water treatment applications [16], [17], [18]. While several studies have explored the effectiveness of biochar for dye adsorption, particularly GM and RB, the comparison between raw biomass and biochar derived from crustacean waste continues to be insufficiently documented [19], [20], [21]. The surface properties of biochar, such as pore distribution, pH of zero charge, as well as kinetic and isotherm studies, are still insufficient to evaluate the performance of these materials [22], [23], [24].

The application of ultrasonic waves generates acoustic cavitation phenomena, characterized by the formation, growth, and violent collapse of microbubbles within the liquid medium. According to the literature, the implosive collapse of cavitation bubbles generates localized extreme conditions, with transient temperatures of approximately 4000–6000 K and pressures reaching several hundred atmospheres, together with microjets and shock waves [25]. These effects significantly reduce the thickness of the boundary layer surrounding the adsorbent particles, improve mass transfer rates, and facilitate the diffusion of adsorbate molecules toward active adsorption sites. In addition, ultrasonic irradiation promotes pore accessibility by removing surface deposits and preventing particle agglomeration, thereby increasing the effective surface area available for adsorption. For binary dye systems such as Malachite Green and Rhodamine B, ultrasound can also mitigate competitive diffusion limitations, accelerating the transport of both dye molecules into the porous structure of the biochar. Consequently, the synergistic combination of adsorption and sonochemical effects often results in higher adsorption capacities, shorter equilibrium times, and improved removal efficiencies compared with conventional adsorption processes.

No study has investigated the sonochemical-assisted simultaneous adsorption of GM and RB onto blue crab shell-derived biochar. In this context, the present study aims to investigate the sonochemically enhanced simultaneous removal of Malachite Green (GM) and Rhodamine B (RB) using biochar and biomass derived from locally available blue crab shell waste. Particular attention was devoted to understanding the role of ultrasonic irradiation in improving adsorption performance and elucidating the underlying sonochemical mechanisms involved in the binary dye removal process. The thermal (TG/dTG), structural (N_2_ adsorption–desorption), morphological (SEM), and surface chemical (pHpzc) properties of the prepared materials were thoroughly characterized to establish the relationship between physicochemical properties and adsorption behavior. Batch adsorption experiments were conducted under both conventional and ultrasonic conditions. Furthermore, the influence of acoustic cavitation on dye diffusion, pore accessibility, and active-site utilization was examined.

## 2 Experimental

### 2.1 Materials

**Fig. 1:**
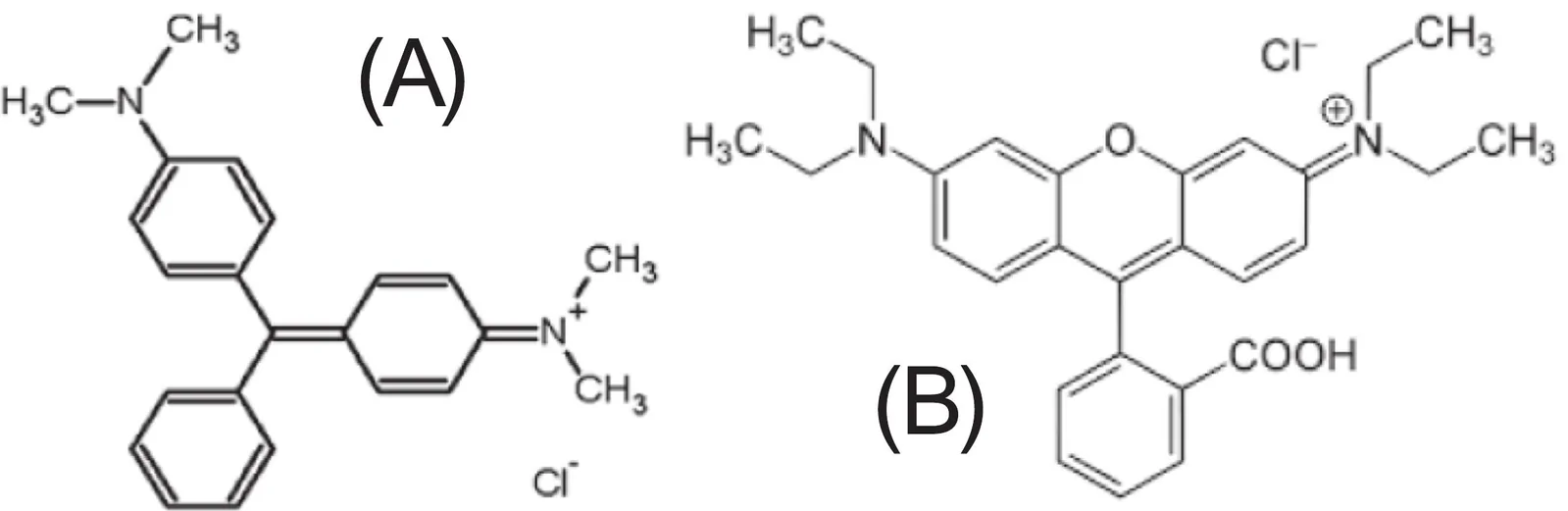
(A): Green Malachite (GM) and (B): Rhodamine B (RB) dyes chemical structures.

The dyes used in this study were Malachite Green (GM) and Rhodamine B (RB), purchased from Sigma–Aldrich with a purity of 99.99%. These two dyes were specifically selected because GM and RB are fluorescent tracers detected/used in the real seawater system. Fig. 1 presents the chemical structures of the studied dyes.

### 2.2 Crab shell preparation

The blue crab shells (BC) were collected from ZaratCrab Seafood Company, Gabes Governorate, Tunisia. The collected shells were thoroughly rinsed several times with tap water followed by distilled water until a neutral pH was reached, following the procedure reported by Wu et al. [26]. The washed shells were then dried in an oven at 105 °C for 24 h to remove residual moisture. After drying, the shells were ground using a knife mill and subsequently sieved to obtain particles with a size below 250 µm. The resulting material was used as the crab shell biomass.

**Fig. 2:**
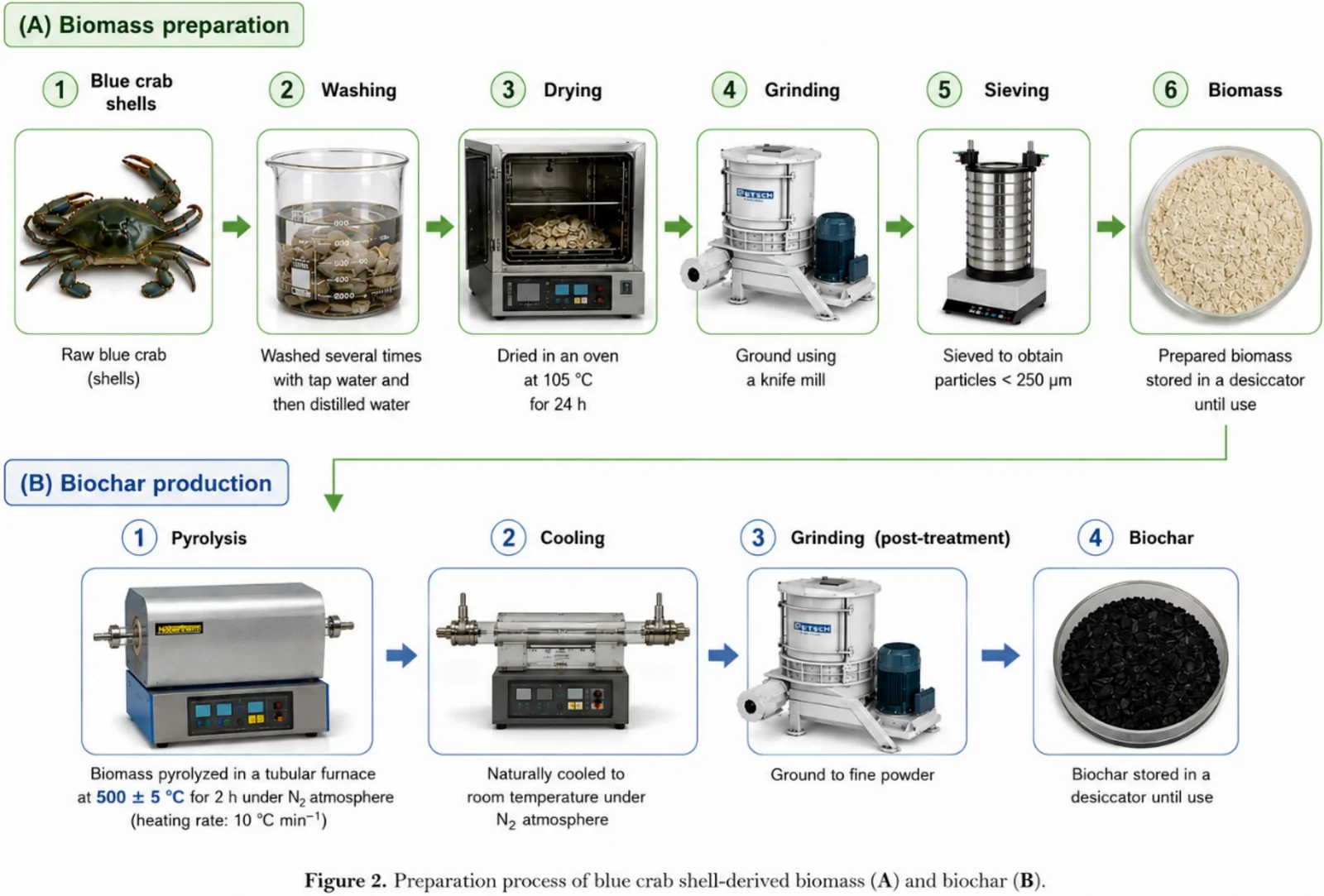
Schema of preparation of crab shell Biomass and its Biochar.

For biochar preparation, the sieved crab shell biomass was subjected to pyrolysis in a tubular furnace under a nitrogen (N_2_) atmosphere to minimize oxygen exposure. The biomass was heated to 500 ± 5 °C and maintained at this temperature for 2 h. After pyrolysis, the material was allowed to cool naturally to room temperature under an N_2_ atmosphere. The resulting biochar was then ground to a fine powder and stored in a desiccator until further use. The overall preparation procedure is illustrated in Fig. 2.

The physical characteristics of the particles were examined using the methodology outlined in our earlier research [20].

### 2.3 Characterization of the crab shell

The thermal gravimetric (TG) analysis utilized a Mettler Toledo TG/DSC STAR thermogravimetric analyzer. Particle size distribution was assessed via laser diffraction using a Coulter LS particle size analyzer from Beckman Coulter, CA. Morphology of the crab shell was examined with a high-resolution compact third-generation SEM, while the HITACHI FlexSEM1000 II. N_2_ adsorption–desorption analyses were conducted using a Quanta-chrome Autosorb-1 automatic volumetric sorption analyzer after 24-hour degassing at 373 K under vacuum. BET specific surface areas of the biomass and its biochars were calculated from these data within the range of relative pressures 0.03–0.30 [27]. The point of zero charge (pH_pzc_) was determined following the method described by Msaadi et al. [28].

### 2.4 Batch adsorption process

0.01 g of BC Biomass (or its Biochar) was added to 50 mL of the GM or RB solutions to test adsorption efficiency of the material [29]. The response parameter was then used to analyze it, and the adsorption yield Y (%) was found using equation Eq.1.

(1) $$Y\left(\%\right)=\frac{C_{\mathrm{ads}}}{C_{0}}\times 100$$

(2) $$q\left(mg/g\right)=C_{\mathrm{ads}}\frac{V}{m}$$

Where $C_{0}$ is the initial dye concentration (mg L^−1^), $C_{f}$ is the final residual dye concentration (mg L^−1^), $C_{\mathrm{ads}}$ is the adsorbed dye concentration (C0-Cf) (mg L^−1^), V is the solution volume (L), and m is the adsorbent mass (g).

The reusability of the biochar adsorbent was evaluated through successive adsorption–desorption cycles. After each adsorption cycle, the biochar was separated by filtration, washed thoroughly with distilled water to remove residual dye, and then dried at 105 °C for 12 hours before reuse. A total of 10 cycles were performed under identical adsorption conditions to assess the stability and regeneration capability of the biochar. Adsorption efficiency was measured after each cycle to monitor any decrease in performance.

### 2.5 Optimization studies

The analysis was carried out using Response Surface Methodology (RSM) with the assistance of STATISTICA Software (Version 12.4, September 2022, United States. Inc.). To model the response variables, quadratic statistical models were developed, incorporating polynomial and interaction terms. A second-order quadratic polynomial model was applied to analyze the experimental data, enabling a deeper understanding of how the operational parameters influenced the outcomes. The model is mathematically expressed as follows (Eq.3):

(3) $$Y=\beta_{0}+\sum_{i=1}^{n}\beta_{i}X_{i}+\sum_{i=1}^{n}\beta_{\mathrm{ii}}{X^{2}}_{i}+\sum_{i<j}^{n}\beta_{\mathrm{ij}}X_{i}X_{j}\pm \Delta y$$

Here, Y represents the dependent variable (response system), specifically the efficiency of GM and RB dye removal. Xi refers to the independent variables under investigation (Factors). The coefficients β_0_  (weighted average of the model), βi (linear coefficients), βii (quadratic coefficients), and βij (interaction coefficients) define the model, while n indicates the total number of variables included in the optimization process. The term Δy accounts for random errors in the data and is called the residual of the model.

Selection of the optimal polynomial model was guided by several criteria, including Pareto chart analysis. The obtained sequential p-values were less than or equal to 0.05, indicating the significance of the model terms, while the lack of fit test resulted in a p-value greater than 0.05, confirming that the model was statistically adequate. The maximization of adjusted and predicted R-squared values ensured that their difference did not exceed 0.2. Non-aliased models were prioritized, and the significance of additional terms was carefully evaluated. The selected model was subsequently validated using Analysis of Variance (ANOVA).

Further refinement of the model involved assessing whether transformations were necessary to satisfy statistical assumptions. Finally, the interplay between the process parameters and their effects on the response variables was interpreted using a regression equation derived from the statistical model. This interpretation was further supported by a combination of Response Surface Methodology (RSM) and Central Composite Design (CCD), incorporating five levels (-δ, -1, 0, +1, +δ) across three factors: contact time, dye concentration, and temperature. Additionally, Artificial Neural Networks (ANN), a data-driven model inspired by neural networks, facilitate distributed and parallel information processing. ANN has been extensively applied in pollution management and modeling analysis.

### 2.6 Ultrasound-assisted adsorption

Ultrasound-assisted adsorption experiments were performed using a SHARP UT-205S ultrasonic bath (SHARP Manufacturing Systems Corporation, Japan). The ultrasonic system operates at a nominal frequency of 40 kHz with a maximum ultrasonic output power of 200 W. The device is equipped with a bolt-clamped ultrasonic transducer and an adjustable ultrasonic output. The ultrasonic power varied between 50 and 200 W, while the optimum power was determined from the cavitation measurements. The unit is equipped with a digital timer adjustable from 1 to 99 min.

The ultrasound-assisted adsorption experiments were conducted under the previously determined optimum adsorption conditions, using 0.2 g of adsorbent in 1 L of dye solution. The ultrasonic power was the only ultrasound parameter varied in this study. Experiments were conducted at 50, 100, 150, and 200 W, while the frequency (40 kHz) and sonication time (79 min) were kept constant. All other adsorption parameters were maintained at the previously determined optimum conditions. The optimum ultrasonic power was selected based on the maximum acoustic cavitation activity.

The temperature of the system was monitored throughout the ultrasonic treatment using a thermometer. After sonification, the residual concentrations of Malachite Green (GM) and Rhodamine B (RB) were determined using a double-beam UV–Vis spectrophotometer (SP-LUV7600, Infitek). The instrument operates over a wavelength range of 190–1100 nm, with a wavelength accuracy of ±0.3 nm. Absorbance measurements were performed at the maximum absorption wavelengths of 618 nm for GM and 554 nm for RB.

**Fig. 3:**
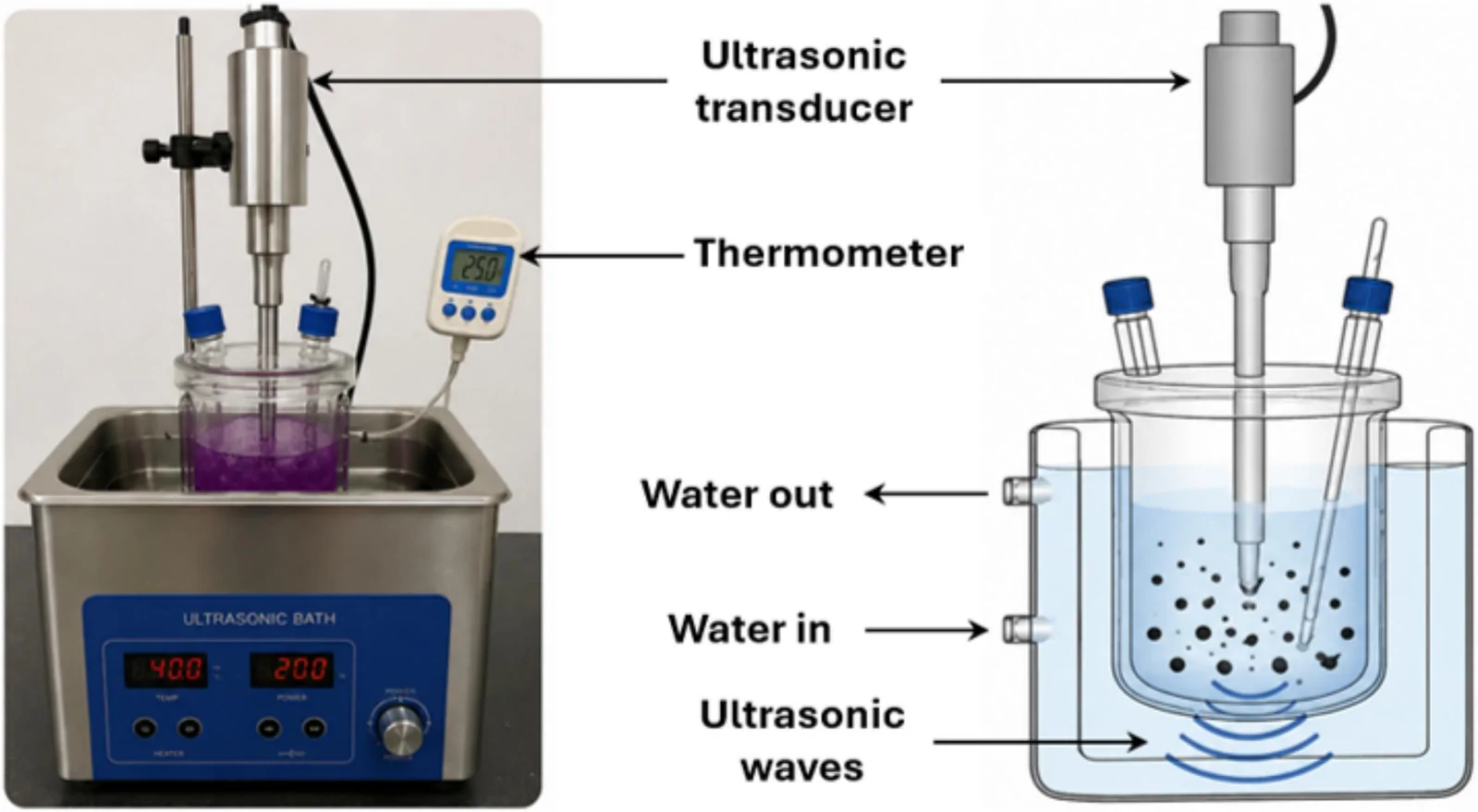
Ultrasound-assisted batch adsorption reactor.

Fig. 3 shows the ultrasonic-assisted reactor used in this study.

## 3 Results and discussion

### 3.1 Composition of the BC biomass and its biochar

The elemental composition of Ca, C, N, S, O, Mg, P, and Cu was determined by EDS analysis. The hydrogen content was estimated by difference from 100% after accounting for the detected elemental composition. The detailed composition of the BC biomass and its biochar is presented in Table 1.

**Table 1:** Element analysis of Blue Crab Biomass and its biochar.

Elements (%)	Ca	C	H	N	S	O	Mg, P, Cu
BC Biomass	28.3	20.8	4.3	2.1	1.1	42.7	∼ 0.7
BC Biochar	19.2	24.1	2.1	1.9	0.9	51.1	∼ 0.7

*H: estimated by difference to 100 %.

Calcium is the main structural element of blue crabs. Indeed, its high concentration in both the raw shell and the biochar stems from the fact that calcium exists in various forms, such as CaCO_3_, calcite or lime, in all types of crabs as mentioned in the literature [30], [31], [32], [33]. The high amount of carbon (around 24.1 %) in the biochar analysis should be noted. This result proves that pyrolysis temperature was complete. Indeed, we should found a decrease in carbon amount after pyrolysis suggest the formation of ash instead of biochar [30], [31], [32], [33]. In our case, the increase from 20 to 28 % of measured carbon content can be explained by the relative concentration effect during pyrolysis. While pyrolysis does cause some loss of volatile organic components and formation of inorganic ash, it also removes oxygen and hydrogen-rich compounds, thereby increasing the relative proportion of fixed carbon in the solid residue. As a result, the carbon content percentage in the biochar often appears higher compared to the raw biomass on a dry basis, even though some carbon is lost as volatile gases [30], [31], [32], [33].

### 3.2 TG/dTG analysis

**Fig. 4:**
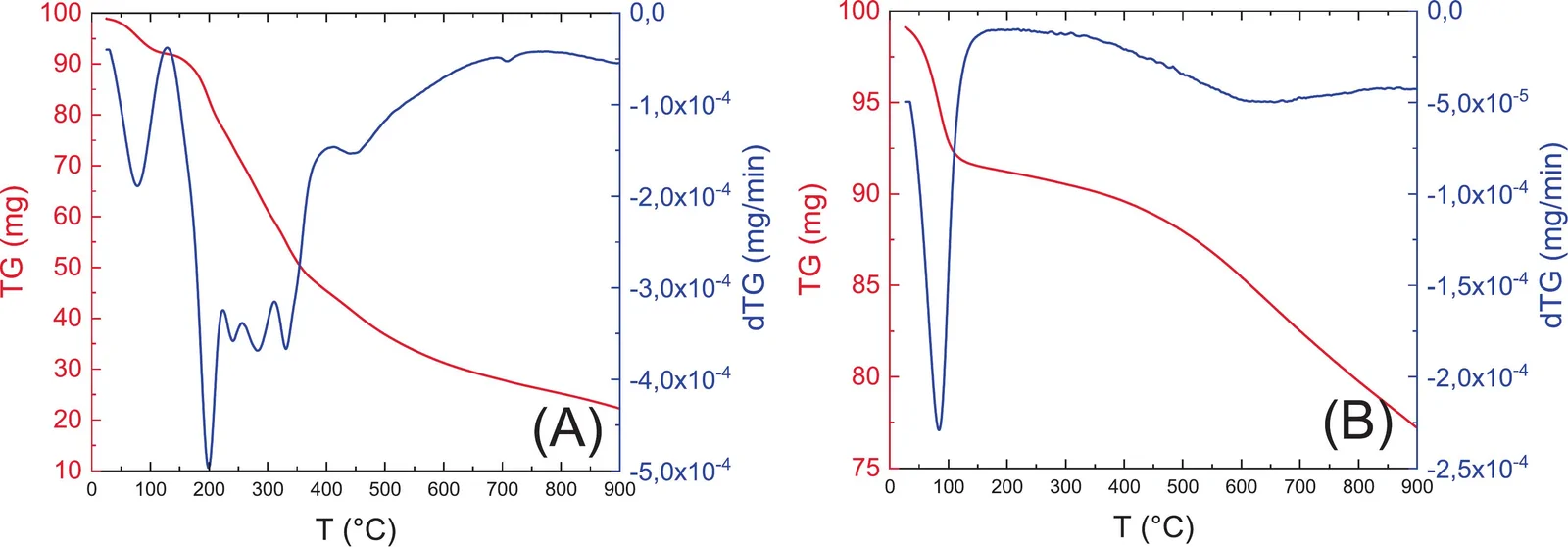
TG/dTG analysis of Blue Crab Biomass and its Biochar.

TG/dTG analysis is a critical factor for successful application of crab shell biomass and its biochar in removing rhodamine B (RB) and green malachite (GM) from seafood wastewater. Fig. 4 illustrates the characteristics of the TG/dTG analysis and the pH at the zero-point charge.

Fig. 4A shows that the dTG curve of BC biomass shows two stages of mass loss. The first occurs between 25-150°C, during which hydration water loss takes place, while the second occurs between 150-550°C (with at least five overlapping processes), which is when the sample decomposes and organic matter pyrolyzes. From 550°C onwards, mass loss slows down. It can also be noted that the first stage is endothermic, while the decomposition process is exothermic. On the other hand, Fig. 4B shows that the dTG curve of the BC Biochar shows two stages of mass loss. The first occurs between 25-140°C, during which hydration water loss takes place, while the second occurs between 150-900°C, which is when the sample decomposes and organic matter pyrolyzes. As for the dTG biomass curve, the first stage is endothermic, while the decomposition process seems to be exothermic. The total mass loss recorded was 76.57 (with 6.88 % of water) and 21.9 % (with 7.46% of water) for the biomass and its Biochar, respectively.

### 3.3 BET study of the BC biomass and its biochar

**Fig. 5:**
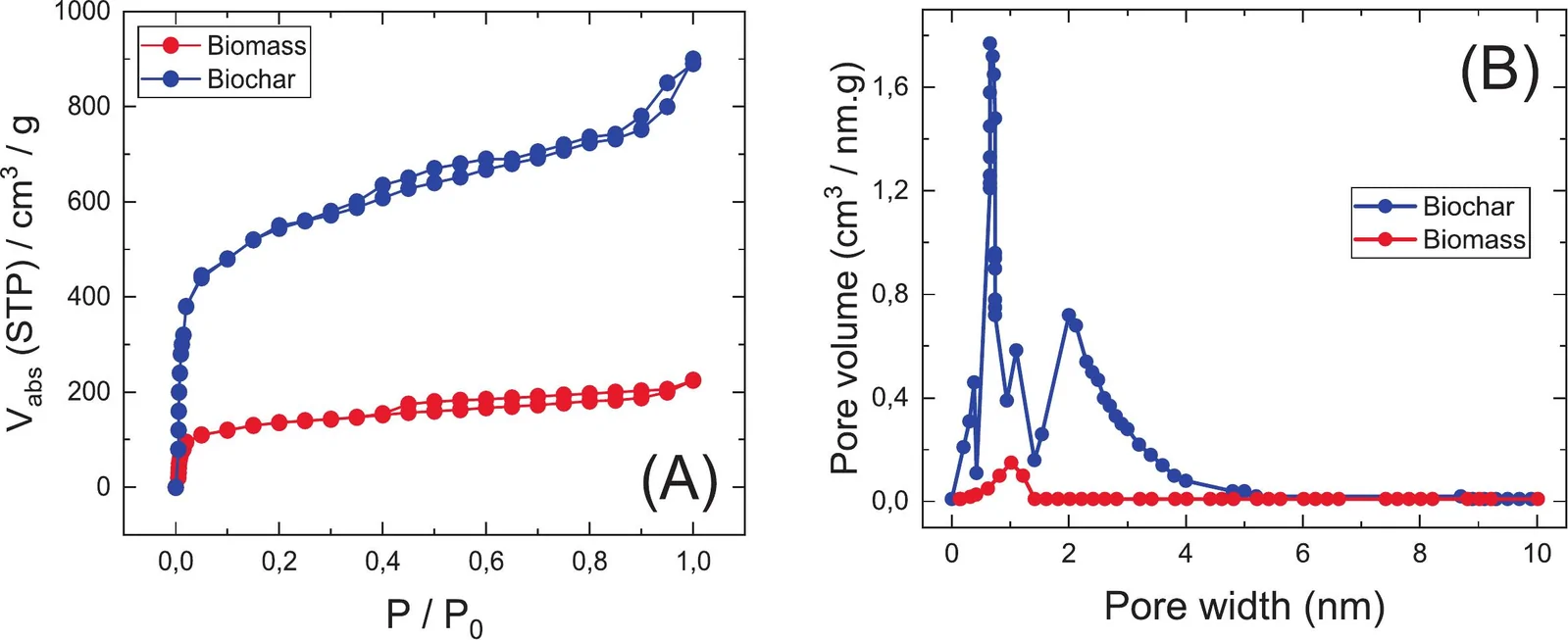
(A) N_2_ adsorption – desorption isotherm profiles (B) Pore distribution for Blue Crab Biomass and its Biochar.

Fig. 5 and Table 2 show the physical properties of Blue Crab Biomass and its Biochar prepared in this work.

**Table 2:** Surface properties of Blue Crab Biomass and its Biochar.

Blue Crab	S_BET_ (m^2^/g)	V_mic_ (cm^3^/g)	V_tot_ (cm^3^/g)	D_av_ (nm)
Biomass	345	0.209	0.102	3.111
Biochar	2217	1.223	0.980	2.056

The pore structure parameters are summarized in Table 2. The biochar had a much higher specific surface area S_BET_ (2217 m^2^/g) than the biomass (345 m^2^/g). The biochar derived from Blue Crab in this study showed a larger BET surface area compared to those from other agricultural waste, such as coals and lignocellulosic precursors [34], [35], [36], suggesting that blue crab was a good precursor for high-surface-area biochar. Average pore sizes recorded were 3.111 nm and 2.056 nm for biomass and biochar, respectively.

In other words, the biochar was primarily composed of micropores, while the biomass was dominated by mesopores. These results can be confirmed by the N_2_ adsorption/desorption isotherms presented in Fig. 5. Indeed, the biochar exhibited type I adsorption isotherms, demonstrating microporous characteristics along with some mesopores. On the other hand, biomass presented type IV isotherms with hysteresis loops at high relative pressures, indicating an abundant presence of mesopores. Furthermore, the biochar displayed a multimodal distribution appearing at around 0.425, 0.625, 0.837, 1.03, 1.26, and 1.40 nm, while the biomass only showed a single peak at around 1.02 nm in the micropore range (Fig. 5), suggesting that pyrolysis conditions were optimal for preparing the biochar with a hierarchical micropore structure.

### 3.4 pH of zero charge

**Fig. 6:**
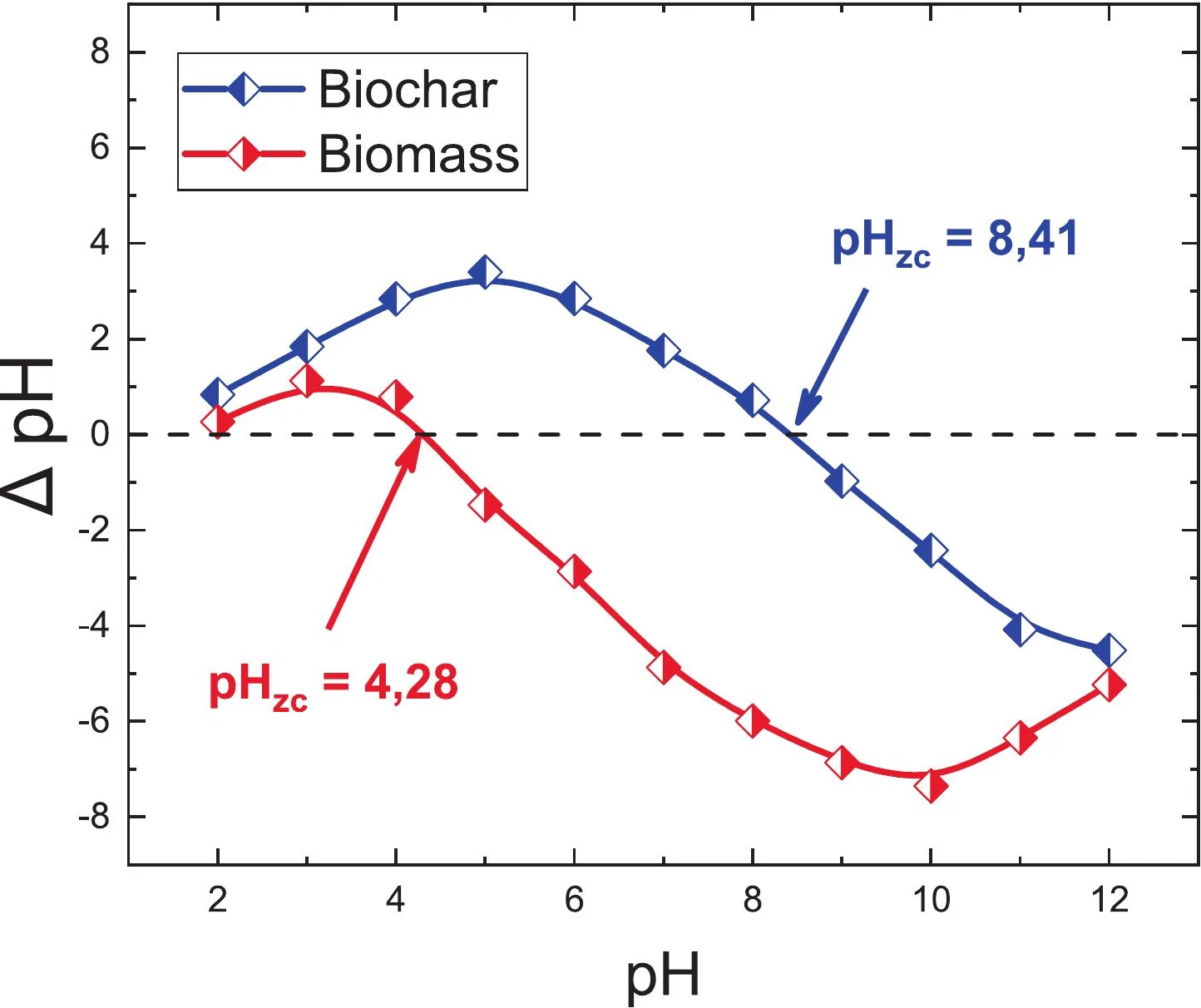
Ph of at the point of zero-charge of the blue crab biomass and its biochar.

The pH at the point of zero charge (pH_pzc_) is the pH value at which a material's surface charge is neutral. In our study, Fig. 6 depicts how the final pH (pH_final_) varies in relation to the initial pH (pH_initial_) during the process.

The pH values at the point of zero charge (pH_pzc_) found were 4.28 and 8.41 for the biomass and its biochar, respectively. This value 4.28 derived from blue crab shells, highlights the surface properties of these derived materials. The biomass, with a relatively low pH_pzc_, exhibits a positive charge below this pH, which promotes adsorption of anionic dyes in acidic conditions. In contrast, the biochar, with a higher pH_pzc_, becomes negatively charged at pH levels below 8.41, allowing it to adsorb cations in alkaline environments. It should be noted that these values are more acid towards the biomass and less alkaline towards the biochar. Indeed, Wu et al. [26] records 4.39 and 9.64 for the BC biomass and biochar, respectively. This can be explained as follows: crab shells are primarily composed of carbon and calcium, and in the case of blue crab shells, they also contain trace amounts of heavy metals such as copper (Cu), zinc (Zn), and magnesium (Mg). The presence of these metals contributes to the alkalinity. Indeed, during production of biochar, elements such as calcium, magnesium, and phosphate present in the biomass are transformed into solid alkaline substances, such as calcium carbonate and phosphate. The increase in pH of the biochar is mainly attributed to these mineral salts, as explained by Choong Jeon [37].

As the GM is anionic dye and the RB is cationic dye, pH bath values will be fixed at 3 and 10 for the GM and RB, respectively.

### 3.5 CCD and ANN optimization

**Fig. 7:**
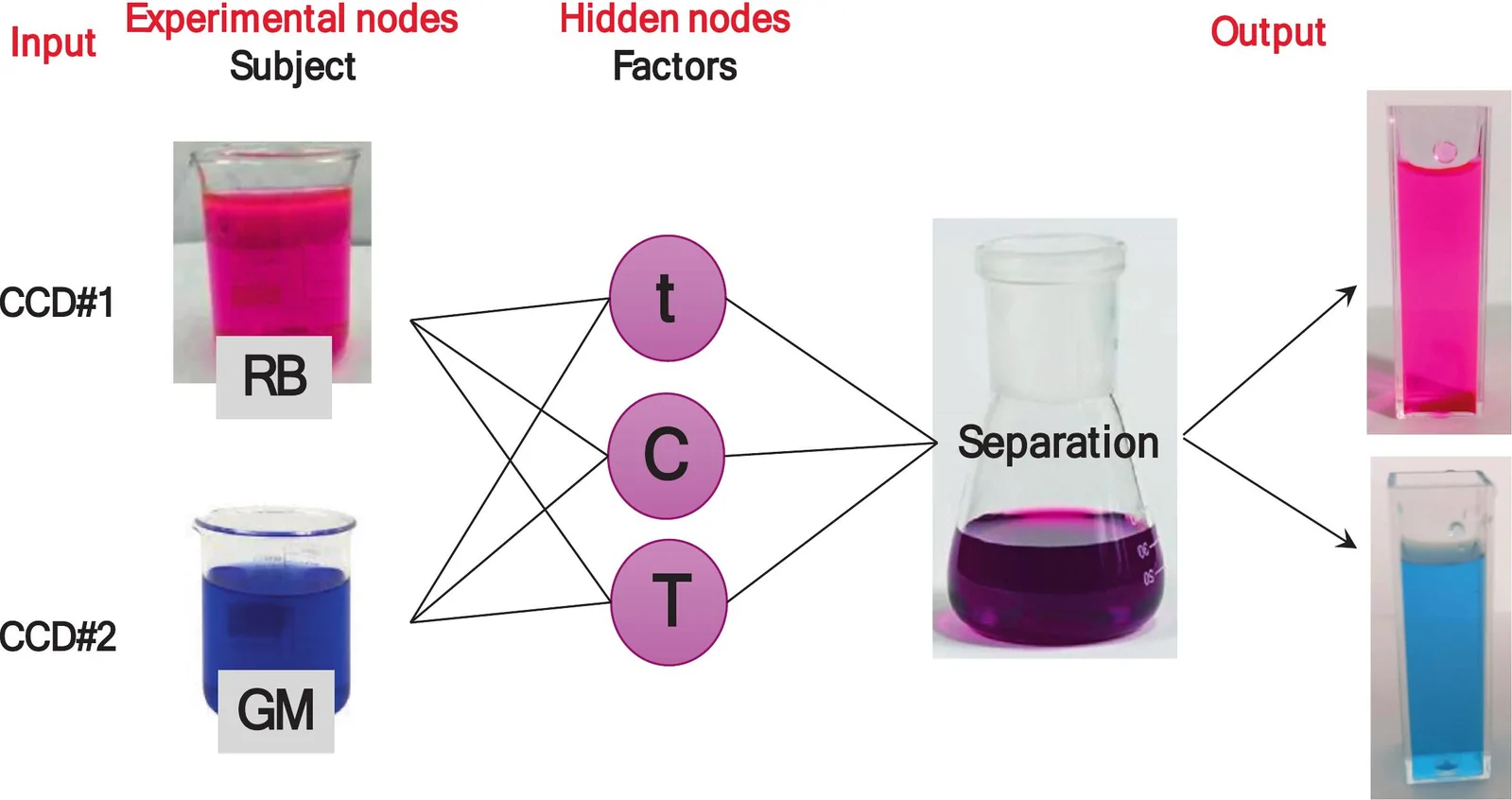
CCD-ANN Architecture of simultaneous/selective removal of RB and GM using BC shell Biomass and its Biochar.

The study utilized Central Composite Design (CCD) and Artificial Neural Networks (ANN) to investigate removal of GM and RB using biomass and biochar derived from blue crab crustaceans (BC). CCD-ANN was employed to optimize the experimental conditions by varying factors such as contact time, dye concentration, and temperature. This combined approach aimed to enhance the efficiency of GM and RB removal, highlighting the potential of crab biomass and its biochar as effective adsorbents. CCD-ANN neuron flow is presented in Fig. 7.

The experimental field shown in Table 3 was a resume from extensive research [30], [31], [32], [33] dealing with dye removal by biomass and its biochar as Blue Crab (BC).

**Table 3:** Experimental field for studied factors.

Factors	Code	(− δ)	(−1)	(0)	(+1)	(+ δ)
Contact time t_c_ (min)	X_1_	1.32	32.00	77.00	122.00	152.68
Dye concentration C (mg/L)	X_2_	6.14	30.00	65.00	100.00	123.86
Temperature media T (°K)	X_3_	294.36	308.00	328.00	348.00	361.64

δ is the code of rotatability and orthogonality for the adopted CCD-ANN design. It is calculated using Eq. (4) [27], [29], [38], [39], [40], [41], [42]:

(4) $$\delta =\sqrt[{4}]{\frac{n_{f}\times {\left(\sqrt{n}-\sqrt{n_{f}}\right)}^{2}}{4}}$$

where n_f_ is the number of runs proposed by the factorial plan (Eq.5), and n is the total number of runs proposed by the CCD model, as explained in Eq. (6) [38]:

(5) $$n_{f}=l^{3}$$

(6) $$n=n_{f}+l\times 3+c\times r$$

Where l is the level, c the number of model centers, and r its number of repetitions. In our case, n and the code δ are 17 runs and 1.69, respectively. Equivalency between design codes and factor values is presented elsewhere.

**Fig. 8:**
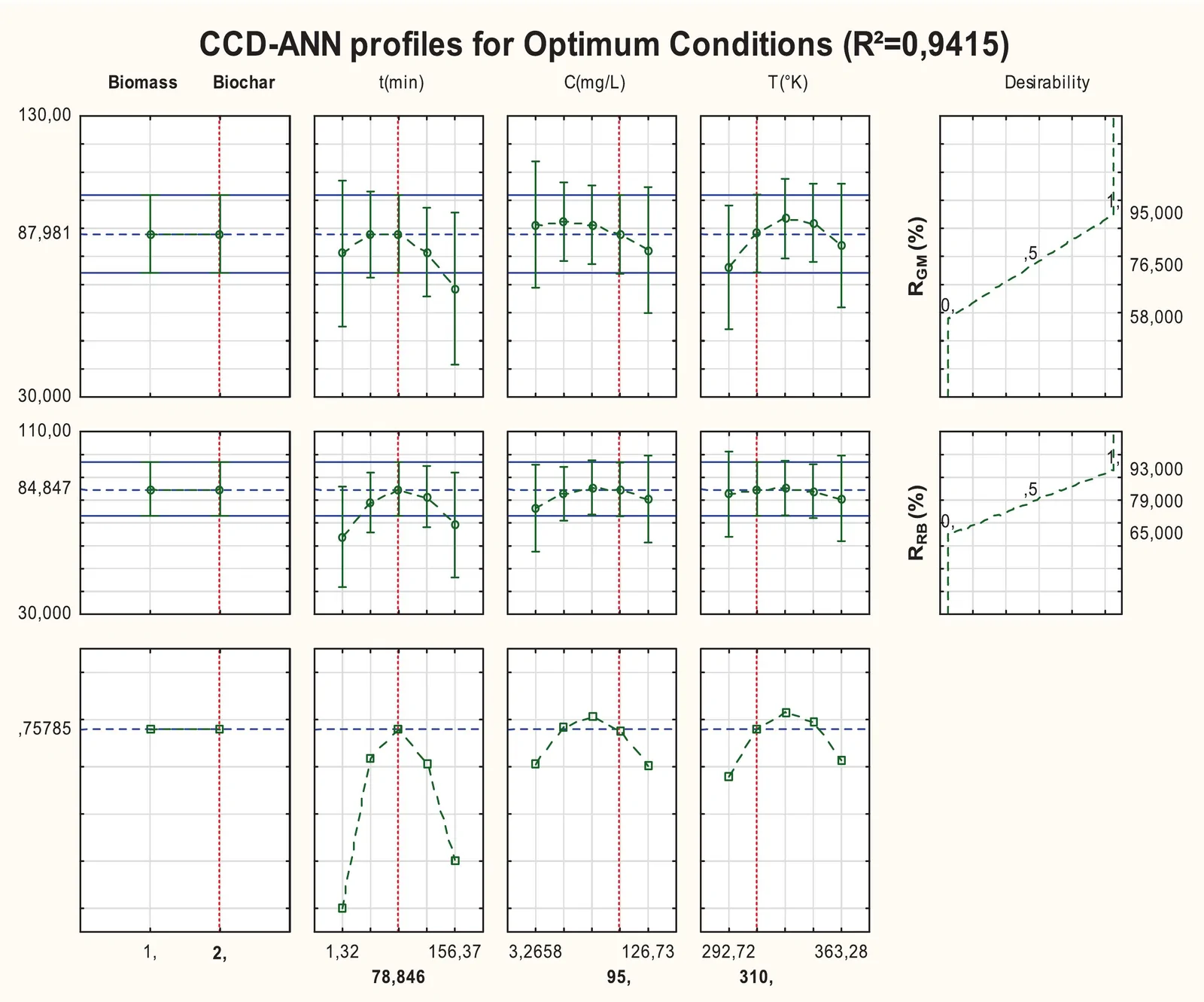
Optimum conditions of GM (pH 3) and RB (pH 10) binary adsorption onto BC Biomass and its Biochar.

The optimum conditions for both RB and GM dyes generated by the software are presented in Fig. 8.

First, the regression coefficient is close to the unit (R^2^ = 0.94), indicating successful multi-optimization for the dyes studied. Experimental data also appear to be accurate, with adsorption yields ranging from 58% to 95% for the GM dye and from 65% to 93% for the RB dye. This suggests that both the BC biomass and its biochar adsorbents have a greater affinity for the anionic dye GM. The optimal conditions identified by the software were a contact time of 79 minutes, a dye concentration of 95 mg/L, and a temperature of 310 K. The high temperature of 310 K, noted as optimal for adsorption of both GM and RB, suggests the endothermic nature of the adsorption process.

Despite the optimal conditions, the CCD-ANN model provided a combined polynomial equation capable of predicting removal efficiency for both GM and RB dyes, known as canonic analysis, and correlating the responses into a single composite metric, as shown in Eq.7:

(7) $$Y=\beta_{0}+\sum_{i=1}^{n}\beta_{i}X_{i}+\sum_{i=1}^{n}\beta_{\mathrm{ii}}{X^{2}}_{i}+\sum_{i<j}^{n}\beta_{\mathrm{ij}}X_{i}X_{j}\pm \Delta y$$

(8) $$\beta_{0,i,ii,ij}\approx \;\omega_{1}\varphi_{1,i,ii,ij}+\omega_{2}\varphi_{2,i,ii,ij}$$

Where ω_1_ and ω_2_ are the artificial neural network weights, and φ_1_ and φ_1_ are the neuron coefficients.

The simulation should be as follows (Eq. 9):

$Y=89.28+1.42X_{1}+0.08X_{1}^{2}-5.55X_{2}+0.02X_{2}^{2}-3.05X_{3}+0.01X_{3}^{2}\pm 0.39$ *(Eq. 9)*

The above equation illustrates the impact of three main factors: time (X_1_), concentration (X_2_), and temperature (X_3_), on the removal of dyes. The linear term of X_1_ (time) has a positive coefficient of 1.42, indicating that increasing time improves dye removal. Additionally, the quadratic term X_1_^2^ with a coefficient of 0.08 suggests that the effect of time becomes more pronounced as time increases, thus indicating that dye removal is favored by longer durations, but in a nonlinear manner. In contrast, the initial concentration of the dye (X_2_) has a negative effect on dye removal, as evidenced by the coefficient of −5.55 for the linear term of X_2_ . This suggests that higher dye concentrations hinder dye removal, possibly due to saturation of the treatment process. However, the quadratic term X_2_^2^ , though positive (0.02), indicates that this saturation effect may diminish after a certain concentration threshold. Regarding temperature (X_3_), the effect is also negative with a coefficient of −3.05 for the linear term, indicating that increasing temperature reduces the effectiveness of dye removal. However, the quadratic term X_3_^2^ , with a positive coefficient of 0.01, suggests that the temperature effect could slightly stabilize or reverse at higher temperatures, though this effect is minimal. Finally, the uncertainty of ± 0.39 indicates that any variation in the model less than this value has no significant effect on dye removal. In other words, variations in the results below 0.39 units can be considered negligible. To conclude, dye removal is influenced in a complex manner by these three factors, and the right balance must be struck between them to optimize the process.

**Fig. 9:**
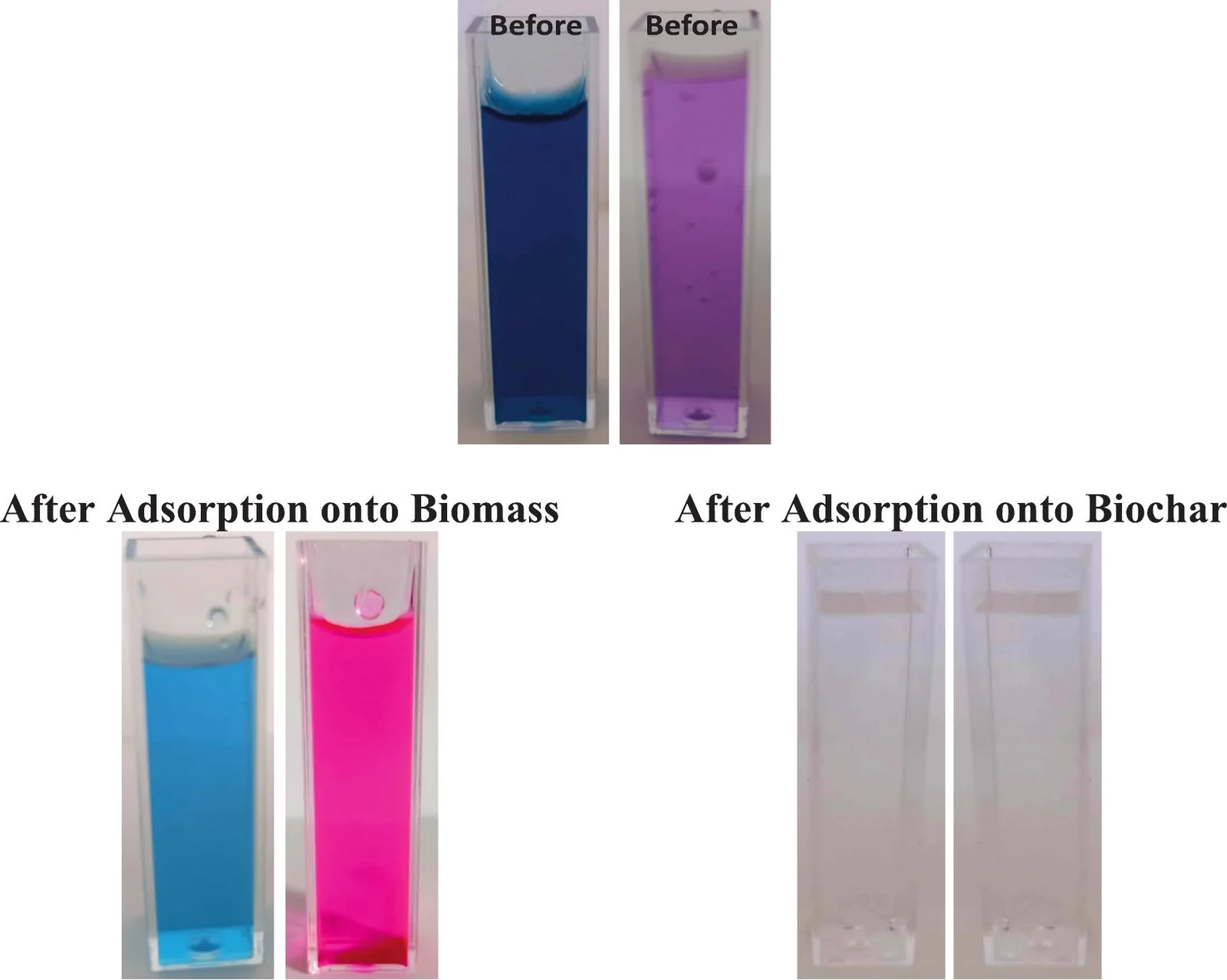
Solutions of GM (pH 3) and RB (pH 10) before and after adsorption onto BC Biomass and its Biochar at optimum conditions (t = 79 min, C = 95 mg/L, and T = 310°K).

**Fig. 10:**
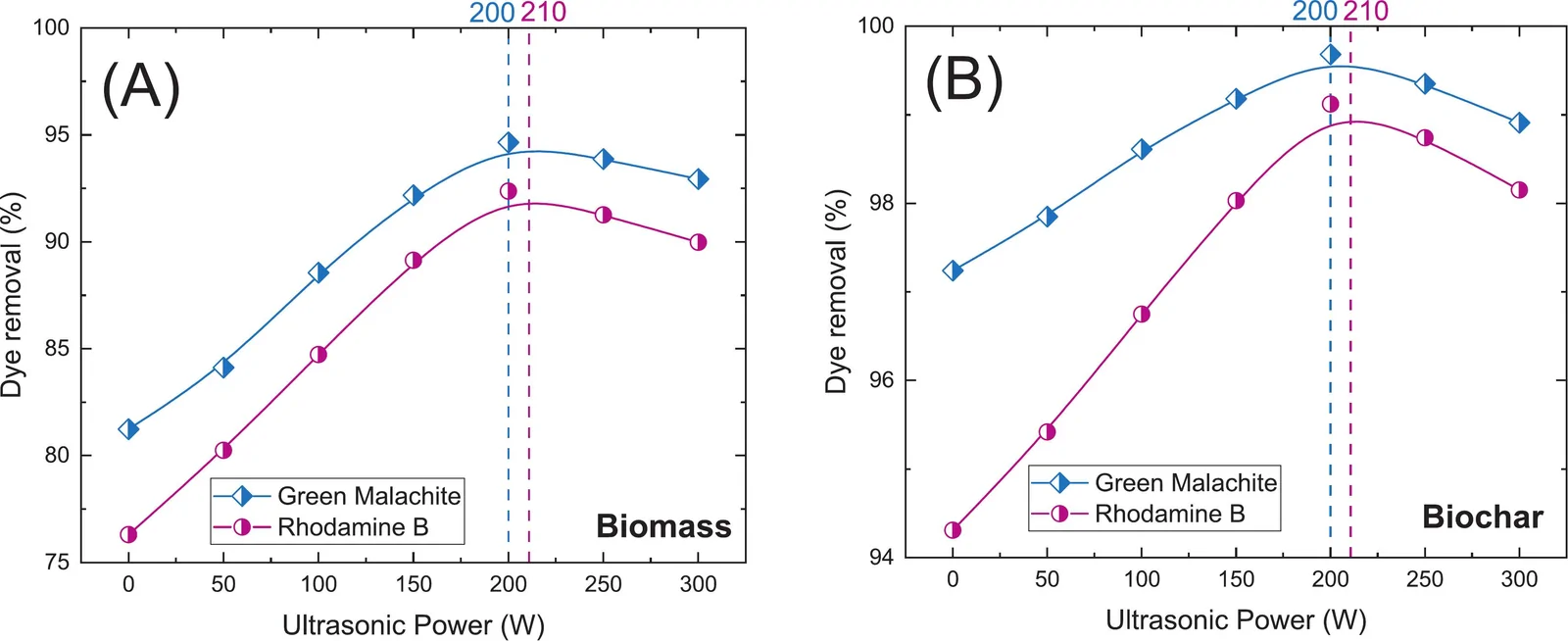
Influence of ultrasonic power on the ultrasound-assisted adsorption of Malachite Green (GM) and Rhodamine B (RB) by blue crab shell (A): Biomass and (B): Biochar under optimized adsorption conditions.

Fig. 9 illustrates the GM and RB solutions before and after adsorption under generated optimum conditions. Fig 10 presents the influence of ultrasonic power on the ultrasound-assisted adsorption of Malachite Green (GM) and Rhodamine B (RB).

Under the optimized conditions, the removal efficiencies achieved by the biomass were 81.24% for GM and 76.31% for RB, whereas biochar exhibited substantially higher removal efficiencies of 97.24% and 94.31% for GM and RB, respectively (Fig.10). The lower removal efficiencies observed for biomass may be attributed to its less developed porous structure and the lower availability of accessible adsorption sites. In the binary system, GM and RB simultaneously compete for the available adsorption sites and pore spaces, and their different molecular characteristics may result in differences in adsorption affinity and accessibility. The higher removal efficiencies obtained with biochar indicate that its developed porous structure provides more favorable sites for the simultaneous uptake of both dyes. As shown in Fig. 9, the solutions treated with biochar became nearly transparent after adsorption, visually confirming the high removal efficiencies obtained for both GM and RB.

### 3.6 Effect of ultrasonic irradiation on the simultaneous adsorption of MG and RB

The ultrasonic irradiation influence on the simultaneous adsorption of both textile dyes was investigated under the optimized adsorption conditions determined previously (79 min, 95 mg/L and 310 K).

For both adsorbents, the dye removal efficiency increased progressively as the ultrasonic power was raised from 0 to 200 W, reaching maximum values of approximately 94.7% and 92.3% for GM and RB, respectively, with the biomass, and 99.6% and 99.1% for GM and RB, respectively, with the biochar. This enhancement can be attributed to the increase in acoustic cavitation intensity, which promotes microstreaming, turbulence, and microjet formation in the liquid phase. These phenomena reduce the thickness of the hydrodynamic boundary layer surrounding the adsorbent particles and facilitate mass transfer of dye molecules toward the active adsorption sites.

The improvement was more pronounced for the biomass than for the biochar. Indeed, the biomass exhibited an increase of approximately 13.5% for GM and 16.0% for RB when the ultrasonic power was increased from 0 to 200 W. In contrast, the biochar showed a more moderate enhancement of about 2.4% for GM and 4.8% for RB over the same power range. This behavior is mainly related to the highly developed porous structure and larger specific surface area of the biochar, which already provides excellent adsorption performance under conventional conditions. Consequently, the relative contribution of ultrasound is more significant for the less porous biomass.

Beyond 200 W, a slight decrease in removal efficiency was observed for both materials and dyes. Excessive ultrasonic power may induce bubble coalescence and acoustic shielding effects, reducing the effective transmission of ultrasonic energy into the solution. Moreover, the formation of large cavitation bubbles decreases the intensity of microjets and limits the beneficial effects of acoustic cavitation on mass transfer. Therefore, an ultrasonic power of 200 W was identified as the optimum operating condition for maximizing simultaneous GM and RB removal.

### 3.7 Sono-chemiluminescence evaluation of acoustic cavitation

**Fig. 11:**
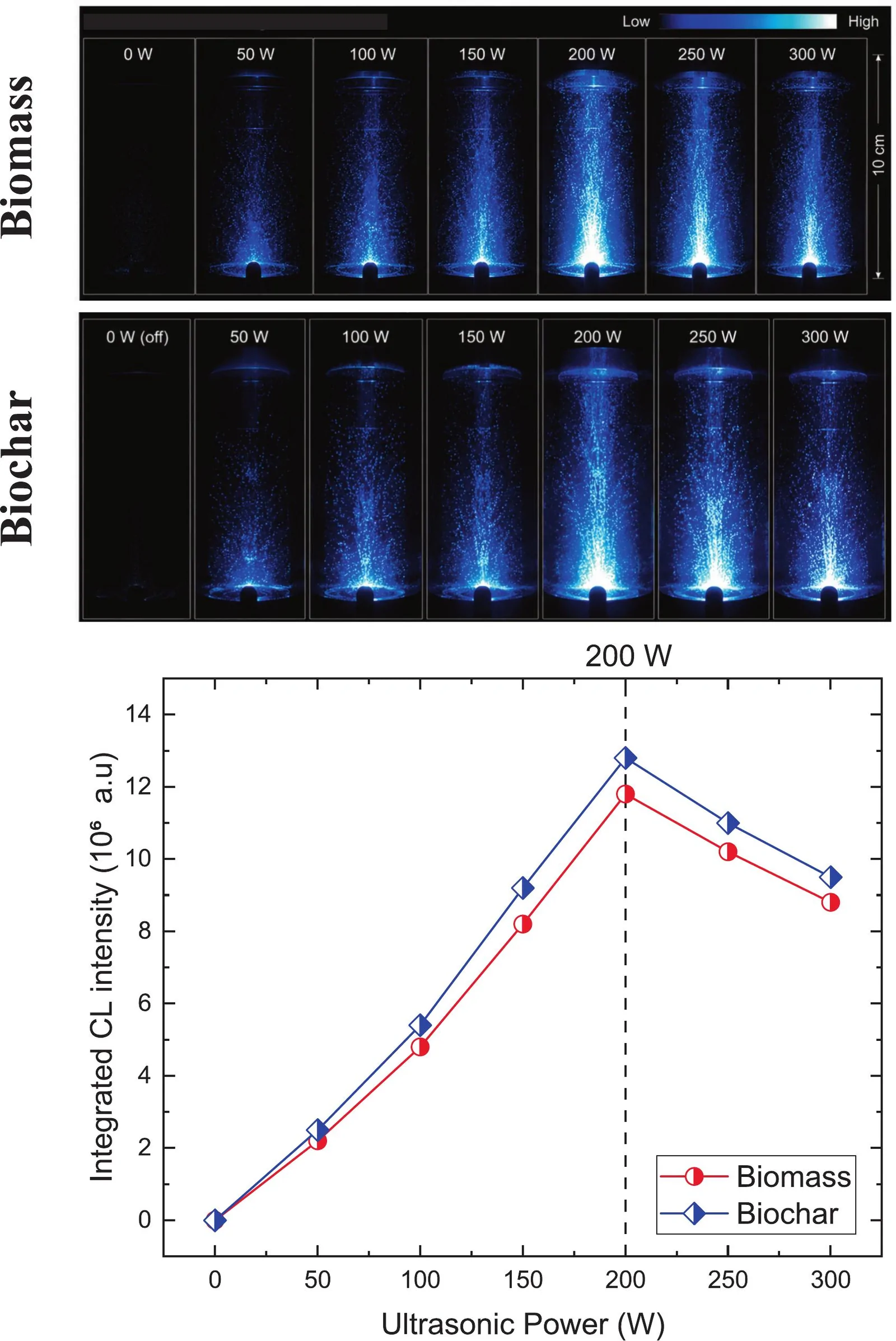
Sono-chemiluminescence (SCL) images and integrated chemiluminescence intensity recorded at different ultrasonic powers after 79 min contact time adsorption process.

Fig. 11 shows the sono-chemiluminescence (SCL) images and integrated chemiluminescence intensity recorded at different ultrasonic powers during the adsorption process at optimum contact time 79 min.

As knowing the sono-chemiluminescence is directly associated with the formation of cavitation bubbles, which generate highly energetic microenvironments and reactive species in the liquid phase. Therefore, the CL intensity can be considered an indirect indicator of cavitation activity.

As shown in Fig. 11, the CL intensity increased progressively with ultrasonic power from 0 to 200 W for both materials. The integrated intensity rose from approximately 2.2 × 10⁶ and 2.5 × 10⁶ a.u. at 50 W to 11.8 × 10⁶ and 12.8 × 10⁶ a.u. at 200 W for biomass and biochar suspensions, respectively. This increase indicates that higher acoustic power promoted the formation of a greater number of cavitation bubbles and intensified their collapse, thereby enhancing cavitation activity within the adsorption medium.

The maximum CL intensity observed at 200 W suggests that this power corresponds to the optimum cavitation condition. Interestingly, the same ultrasonic power also yielded the highest removal efficiencies for both Malachite Green and Rhodamine B, highlighting the close relationship between cavitation intensity and adsorption enhancement. The increased cavitation activity likely generated stronger microstreaming, shock waves, and microjets, which improved mass transfer and facilitated the transport of dye molecules toward the adsorption sites.

Beyond 200 W, the CL intensity decreased for both systems despite the increase in nominal ultrasonic power. This behavior is commonly attributed to excessive cavitation leading to bubble coalescence and acoustic shielding effects. Under such conditions, large bubble clouds absorb and scatter part of the ultrasonic energy, reducing the number of effective bubble collapses and consequently lowering cavitation efficiency.

Nevertheless, the difference between biomass and biochar remained relatively small compared with the substantial difference in adsorption performance. This observation suggests that the superior dye removal achieved by biochar is primarily governed by its highly developed porous structure and large specific surface area, while acoustic cavitation mainly acts as a mass-transfer intensification mechanism. Consequently, the adsorption enhancement results from a synergistic interaction between cavitation-induced transport phenomena and the intrinsic adsorption properties of the biochar.

### 3.8 Energy Efficiency Assessment

To evaluate the practical relevance of ultrasound-assisted adsorption, the energy requirement and the enhancement induced by acoustic cavitation were assessed under the optimum operating conditions (200 W, 79 min, 1 L, 0.2 g adsorbent).

The performance of the process was analyzed in terms of specific energy consumption (SEC), cavitation enhancement index (CEI), and adsorption enhancement achieved after ultrasonic treatment.

The equations are as follows (Eq. 9-11) :

(9) $$\mathrm{SEC}=\frac{P\times t}{V}$$

Where P is the ultrasonic power (W), t is the treatment time (h) and V is the treated solution volume (L).

(10) $$\mathrm{CEI}=\frac{CL_{\mathrm{opt}(200W)}}{CL_{\mathrm{ref}(50W)}}$$

Where $CL_{\mathrm{opt}}$ is the sonochemiluminescence intensity measured at the optimum ultrasonic power (200 W) and $CL_{\mathrm{ref}}$ is the sonochemiluminescence intensity measured at the reference ultrasonic power (50 W).

(10) $$\mathrm{AEF}=\frac{Y_{\mathrm{US}}-Y_{0}}{Y_{0}}\times 100$$

Where AEF is the adsorption enhancement percentage, Y_US_ is the adsorption efficiency obtained under ultrasonic irradiation (%), Y_0_ is the adsorption efficiency obtained under conventional adsorption conditions (%).

The corresponding parameters are summarized in Table 4.

**Table 4:** Experimental field for studied factors.

**Parameter**	**Biomass**	**Biochar**
Optimal ultrasonic power (W)	200	200
Maximum CL intensity (×10⁶ a.u.)	11.8	12.8
Treatment time (min)	79	79
Specific Energy Consumption (kWh L^−1^)	0.263	0.263
GM removal without US (%)	81.2	97.2
GM removal with US (%)	94.6	99.6
Adsorption Enhancement Factor (GM)	1.17	1.03
RB removal without US (%)	76.3	94.3
RB removal with US (%)	92.3	99.0
Adsorption Enhancement Factor (RB)	1.21	1.05
Cavitation Enhancement Index (CEI)	5.36	5.12

The maximum sono-chemiluminescence intensity reached 11.8 × 10⁶ and 12.8 × 10⁶ a.u. for biomass and biochar, respectively, confirming strong cavitation activity in both systems. Despite similar specific energy consumption (0.263 kWh L^−1^) and comparable cavitation enhancement indices (CEI ≈ 5), the adsorption enhancement induced by ultrasound was significantly higher for biomass than for biochar. The adsorption enhancement factors reached 1.17 and 1.21 for GM and RB removal by biomass, compared with only 1.03 and 1.05 for biochar. These results indicate that biomass adsorption was initially more limited by external and intraparticle mass-transfer resistance, which was effectively reduced by acoustic cavitation. On the other hand, the highly porous structure and large surface area of biochar already ensured efficient adsorption under batch adsorption conditions, resulting in a smaller relative improvement upon ultrasonic irradiation. Therefore, the main effect of ultrasound seems to be associated with cavitation-induced mass-transfer enhancement rather than with the creation of new adsorption sites.

It should be noted that Hamdaoui et al. [43] reported no noticeable degradation of Malachite Green under ultrasonic irradiation at 40 kHz and 9 W, even after 5 h of sonication, indicating that GM was not appreciably sonolyzed under these relatively mild conditions. In contrast, Rhodamine B is more susceptible to direct sonolytic degradation under appropriate ultrasonic conditions. Thus, ultrasound may contribute to dye removal through both cavitation-enhanced adsorption and direct sonolysis, particularly for RB. Accordingly, the present study considers ultrasound as an intensification process rather than attributing dye removal exclusively to adsorption.

### 3.9 SEM analysis

**Fig. 12:**
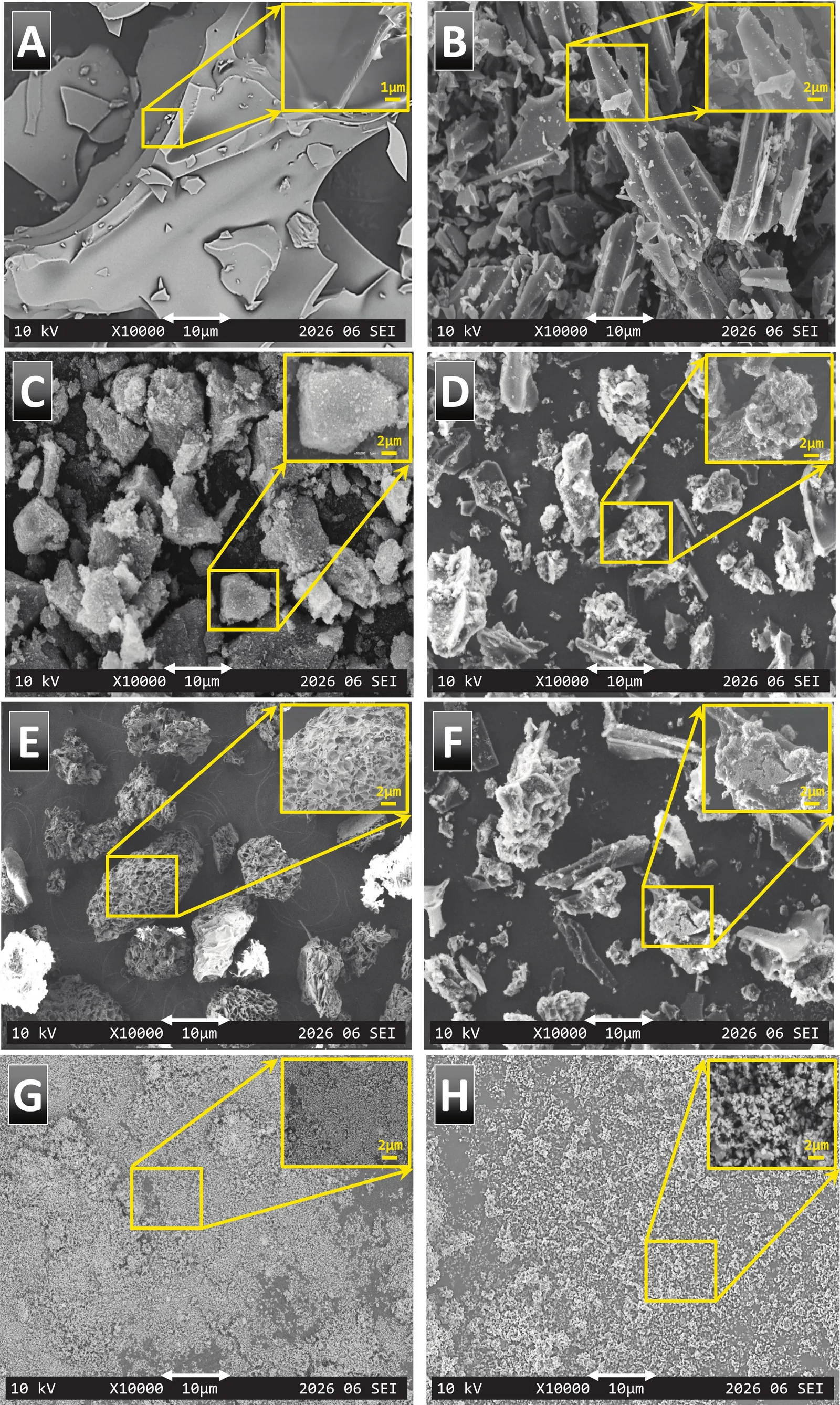

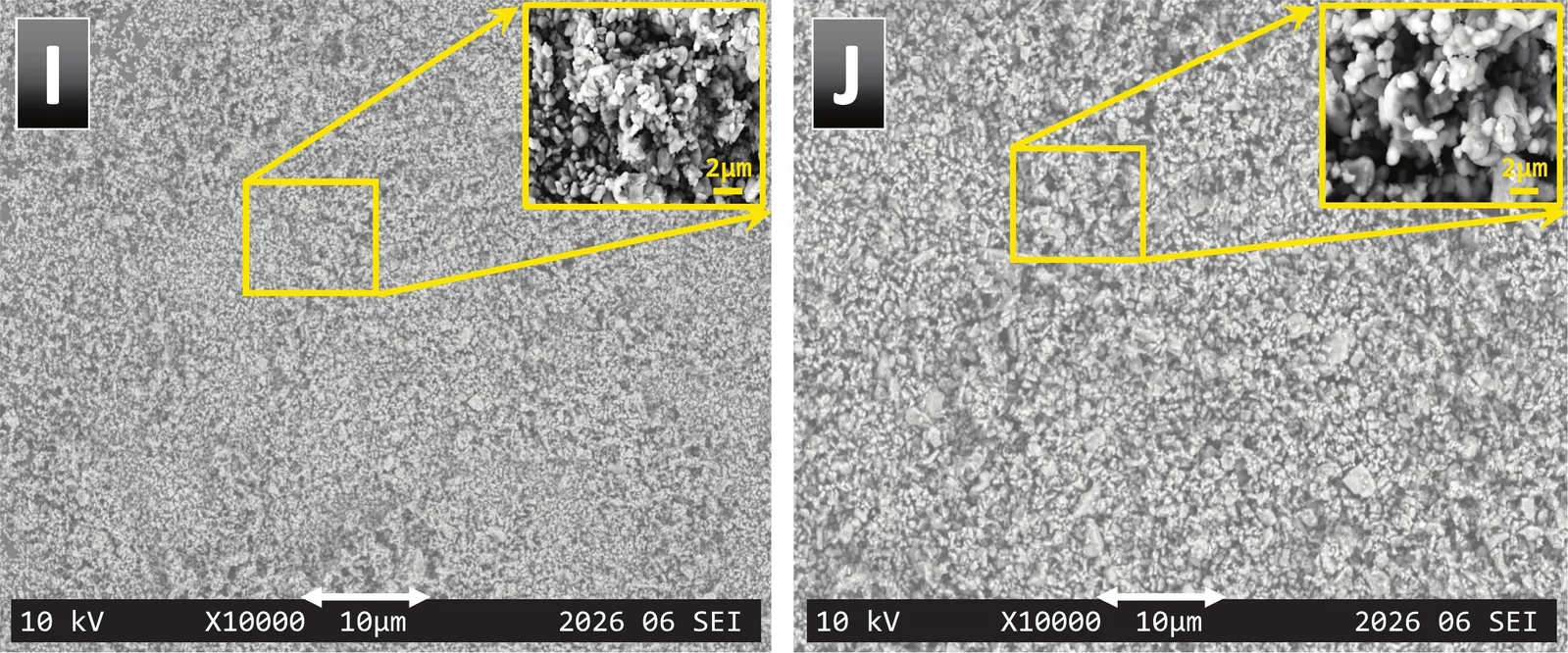
SEM analysis of (A): BC biomass, (B): BC biochar, (C, D) BC biomass and its Biochar after adsorption of GM (pH = 3), (E, F): BC biomass and its Biochar after adsorption of RB (pH = 10), (G,H): BC biomass and its Biochar after Ultrason-assisted adsorption of GM (pH = 3), (I,J): BC biomass and its Biochar after Ultrason-assisted adsorption of RB (pH = 10),

Fig. 12 shows the SEM morphology of the BC Biomass and its biochar before and after removal of GM and RB dyes from aqueous solutions.

The SEM images illustrate the surface characteristics and material modifications at different stages, highlighting the efficiency of blue crab biomass and biochar for the adsorption of GM and RB dyes. The raw biomass (Fig. 11A) exhibits a relatively smooth and homogeneous structure, typical of organic materials such as chitin, but with a limited surface area for adsorption. In contrast, the biochar (Fig. 11B), obtained through pyrolysis, reveals a porous and fibrous structure, significantly increasing the specific surface area and adsorption capacity. After adsorption, the biomass (Figs. 11C and E for GM and RB, respectively) shows dye deposits on its surface, suggesting interaction with specific functional groups naturally present. However, the surface structure appears to saturate quickly, confirming the adsorption limitations of the raw biomass. Conversely, the biochar after adsorption (Figs. 11D and F for GM and RB, respectively) exhibits partially filled pores with clearly visible dye deposits, indicating enhanced adsorption capacity due to its porosity and extended surface area. These observations confirm that converting biomass into biochar significantly improves its adsorption properties, making it a more effective material for treating wastewater contaminated with dyes such as GM and RB. Although post-cycling SEM analyses were not performed in this study, the stability of the biochar is supported by consistent adsorption performance over multiple cycles, in agreement with previous studies reporting the structural integrity and reusability of porous biochars under similar conditions [44].

In contrast, after ultrasonic treatment (Figures G–J), both biomass and biochar surfaces were covered by finer and more uniformly distributed granular deposits. This morphological evolution indicates that acoustic cavitation effectively disrupted particle agglomeration and enhanced the dispersion of adsorbed dye species. The cavitation collapse bubbles generated microjets, shock waves, and microstreaming phenomena, which reduced external mass-transfer resistance and facilitated dye transport toward accessible adsorption sites. Consequently, more homogeneous surface coverage was achieved for both GM and RB. The effect was particularly pronounced for biochar, which exhibited a denser and more compact adsorbed layer than biomass, reflecting a more efficient utilization of its highly developed porous structure. These observations are in good agreement with the sono-chemiluminescence results and confirm that ultrasonic irradiation enhances both external and intraparticle diffusion processes, thereby improving the overall adsorption performance of biomass and especially biochar toward GM and RB removal.

### 3.10 Proposed sono-chemical mechanism

**Fig. 13:**
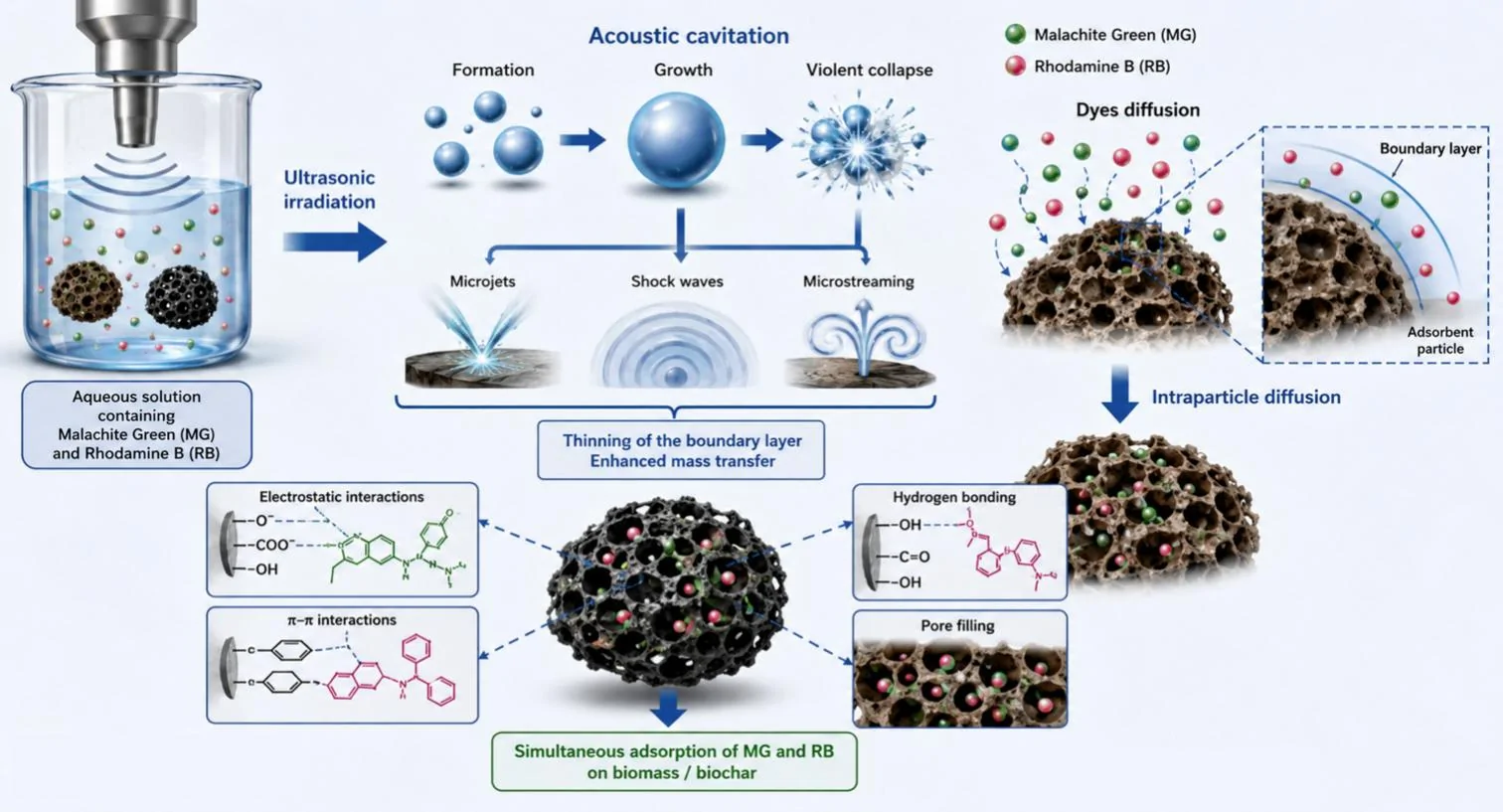
Proposed sono-chemical mechanism for the simultaneous adsorption of GM and RB onto blue crab shell-derived biomass and biochar under ultrasonic irradiation.

Fig. 13 illustrates the proposed mechanism governing the ultrasound-assisted adsorption of Green Malachite and Rhodamine B onto biomass and biochar.

Under ultrasonic irradiation, acoustic cavitation induces the formation, growth, and collapse of cavitation bubbles within the liquid medium. Bubble collapse generates microjets, shock waves, shear forces, and acoustic streaming, which can disturb the hydrodynamic boundary layer surrounding the adsorbent particles and enhance liquid–solid mass transfer. This interpretation is consistent with several recent ultrasound studies. Zhang et al. [45] demonstrated that ultrasonic pretreatment modified the textural properties of chitin-derived biochar and improved its adsorption performance, highlighting the contribution of cavitation-induced mechanical effects to adsorbent accessibility. Lee et al. [46] similarly showed that ultrasonic treatment reduced particle aggregation and increased surface accessibility through cavitation-induced shear effects. Xing et al. [47] further demonstrated that ultrasonic irradiation could improve interfacial transport by modifying concentration polarization and surface accumulation during membrane separation.

More directly related to adsorption, Xu et al. [48] demonstrated that cavitation-induced microjets and shear forces could restructure the pore architecture of activated carbon. Their optimized ultrasonic treatment produced an activated carbon with a specific surface area of 986.13 m^2^ g^−1^ and increased bilirubin adsorption capacity by 16.1% compared with untreated activated carbon. These findings support the possibility that ultrasonic cavitation can improve not only hydrodynamic transport but also the accessibility and connectivity of pores within porous adsorbents. Conversely, Zhu et al. [49] showed that the effect of ultrasound depends strongly on the reactor configuration: in their fixed-bed adsorption system, ultrasonication moderately intensified bulk convection and dispersion but did not significantly improve the overall adsorption–desorption efficiency.

In the present study, the ultrasonic treatment is therefore expected to facilitate the transport of GM and RB molecules from the bulk solution toward the adsorbent surface by reducing external mass-transfer resistance. This interpretation is supported by the coincidence between the maximum sono-chemiluminescence response and the highest adsorption efficiencies obtained at 200 W. At this condition, GM and RB removal reached 94.6% and 92.3% for biomass, compared with 81.24% and 76.31%, respectively, under non-sonicated optimized conditions. Thus, ultrasound increased the removal efficiencies by 13.36 and 16.01 percentage points, corresponding to relative enhancements of approximately 16.4% and 21.0% for GM and RB, respectively. For biochar, the corresponding increases were smaller, from 97.24% to 99.6% for GM and from 94.31% to 99.0% for RB, corresponding to relative enhancements of approximately 2.4% and 5.0%. The stronger relative ultrasonic enhancement observed for biomass suggests that cavitation-induced hydrodynamic effects are particularly important when external mass-transfer limitations are initially more pronounced.

The effect of ultrasound on intraparticle diffusion, however, should be interpreted cautiously. Zhu et al. [49] showed that ultrasound can modify convection and interfacial mass transfer without necessarily enhancing intraparticle diffusion in a fixed-bed configuration. This contrasts with the findings of Xu et al. [48], where ultrasonic treatment modified pore architecture and improved the accessibility of adsorption-relevant pore domains. These apparently different behaviors emphasize the importance of reactor configuration, ultrasonic intensity, adsorbent structure, and operating conditions. In the present batch system, the more homogeneous dye distribution observed by SEM after sonication, together with the enhanced adsorption performance and maximum cavitation activity at 200 W, supports improved adsorbate transport and accessibility of adsorption sites rather than providing direct quantitative evidence of enhanced intraparticle diffusion.

Once GM and RB molecules reach the accessible adsorption sites, their retention may involve electrostatic interactions, hydrogen bonding, π–π interactions, and pore-filling mechanisms. The higher adsorption performance of biochar is mainly attributed to its more developed porous structure and greater availability of accessible adsorption sites. Overall, the combined sono-chemiluminescence, adsorption, and SEM results support a cavitation-assisted mass-transfer mechanism, in which acoustic cavitation enhances fluid motion, reduces interfacial transport resistance, and improves access to the available adsorption sites and pore network.

## 4 Conclusion

To conclude, this study demonstrates the effectiveness of locally sourced crustacean waste biomass and its biochar for the removal of green malachite (GM) and rhodamine B (RB) dyes. Thermal analysis (TG/dTD) reveals distinct mass loss stages for the biomass and biochar, with biochar showing lower total mass loss, indicating its enhanced stability. Adsorption studies, including N_2_ isotherms, show that biochar is primarily microporous, while biomass is mesoporous, with biochar exhibiting optimal pyrolysis conditions for a hierarchical micropore structure. CCD optimization results further highlight the superior dye removal efficiency of biochar, achieving 97.24% and 94.31% removal of GM and RB, respectively, compared to 81.24% and 76.31% for biomass.

Furthermore, ultrasound-assisted adsorption markedly improved dye removal through acoustic cavitation. Sono-chemiluminescence results revealed maximum cavitation activity at 200 W, corresponding to the highest adsorption efficiencies. Under these conditions, dye removal increased to 94.6% and 92.3% for GM and RB using biomass, and to 99.6% and 99.0% using biochar. SEM observations confirmed enhanced mass transfer and more homogeneous dye distribution on the adsorbent surfaces. These findings demonstrate the synergistic effect between ultrasonic cavitation and adsorption, particularly for biochar-based systems.

## CRediT authorship contribution statement

**Najia Hamrouni:** Formal analysis, Conceptualization. **Najla Ben Ameur:** Methodology, Investigation. **Noureddine Allouche:** Supervision. **Jean-Yves Hihn:** Writing – review & editing, Supervision. **Wafa Sassi:** Writing – original draft, Methodology.

## Declaration of competing interest

The authors declare that they have no known competing financial interests or personal relationships that could have appeared to influence the work reported in this paper.
